# The Cost-Effectiveness of AI-Assisted Colonoscopy as a Primary or Secondary Screening Test in a Population-Based Colorectal Cancer Screening Program: Markov Modeling–Based Cost Effectiveness Analysis

**DOI:** 10.2196/67762

**Published:** 2025-09-26

**Authors:** Martin CS Wong, Junjie Huang, Thomas YT Lam, Louis HS Lau, Philip WY Chiu

**Affiliations:** 1 JC School of Public Health and Primary Care Faculty of Medicine The Chinese University of Hong Kong Hong Kong China (Hong Kong); 2 The Chinese Academy of Medical Sciences and Peking Union Medical Colleges Beijing China; 3 School of Public Health Peking University Beijing China; 4 School of Public Health Fudan University Shanghai China; 5 Centre for Health Education and Health Promotion Faculty of Medicine The Chinese University of Hong Kong Hong Kong China (Hong Kong); 6 School of Nursing Faculty of Medicine The Chinese University of Hong Kong Hong Kong China (Hong Kong); 7 Department of Medicine and Therapeutics Faculty of Medicine The Chinese University of Hong Kong Hong Kong China (Hong Kong); 8 Department of Surgery Faculty of Medicine The Chinese University of Hong Kong Hong Kong China (Hong Kong)

**Keywords:** colorectal cancer, screening, fecal immunochemical tests, colonoscopy, artificial intelligence, AI, cost-effectiveness, life years saved

## Abstract

**Background:**

Colorectal cancer (CRC) is the third most common cancer worldwide and poses a heavy burden on health care systems. Early screening for CRC through colonoscopy can effectively reduce both the incidence and mortality associated with CRC. However, the sensitivity of conventional colonoscopy is limited by the level of experience of physicians. Recently, artificial intelligence (AI)–assisted colonoscopy has been shown to have higher sensitivity in detecting CRC and mitigating the limitations concerning physician experience, but few studies have evaluated the cost-effectiveness of AI-assisted colonoscopy in CRC screening.

**Objective:**

This study aimed to evaluate the cost-effectiveness of various CRC screening strategies, including no screening, fecal immunochemical test (FIT) positive result followed by a conventional colonoscopy, FIT positive result followed by AI-assisted colonoscopy, direct colonoscopy, and direct AI-assisted colonoscopy.

**Methods:**

This study modeled a hypothetical population based on current clinical practice in Asia, where CRC screening typically begins at the age of 50 years. The cost-effectiveness of various population-based CRC screening strategies, including AI-assisted colonoscopy, was evaluated by comparing incremental cost-effectiveness ratios (ICERs) and outcome measures such as cancer-related life years lost, number of CRC cases prevented, life years saved, and total cost per life year saved. Data from the international literature and the government gazette were accessed to calculate relevant cost and performance estimates. The data were entered into a decision analysis algorithm based on a Markov model.

**Results:**

Compared to no screening strategy, the ICERs of FIT+colonoscopy (FIT followed by conventional colonoscopy if the FIT result is positive), FIT+AI-assisted colonoscopy (FIT followed by AI-assisted colonoscopy if the FIT result is positive), colonoscopy alone, and AI-assisted colonoscopy were US $138,539, US $122,539, US $203,929, and US $180,444, respectively. When compared with FIT+colonoscopy, the FIT+AI-assisted colonoscopy strategy resulted in fewer cancer-related life years lost (5355 y vs 5327 y), a higher number and proportion of CRC cases prevented (120 vs 132 and 3.7% vs 4.1%), more life years saved (280 y vs 308 y), and lower total cost per life year saved (US $944,008 vs US $854,367). FIT+AI-assisted colonoscopy, which had the lowest ICER (US $122,539) dominated all other strategies, particularly compared to FIT+colonoscopy, with an ICER of –US $36,462. Among primary screening methods, AI-assisted colonoscopy dominated conventional colonoscopy (ICER –US $39,040).

**Conclusions:**

For an Asian population, FIT followed by AI-assisted colonoscopy represented the most cost-effective CRC screening strategy. It had the lowest ICER and the lowest additional cost among all 4 evaluated strategies.

## Introduction

### Background

Colorectal cancer (CRC) constitutes a substantial proportion of disease burden and was the third most common cancer worldwide in 2022 [1,2]. The overall prevalence rates remain high worldwide, with adenomas at 23.9% (95% CI 22.2%-25.8%), advanced adenomas at 4.6% (95% CI 3.8%-5.5%), and CRC at 0.4% (95% CI 0.3%-0.5%) [3]. Over the past decade, there has been an increasing trend of CRC incidence in Asian countries, which necessitates more intensive preventive initiatives [4]. A recent study revealed that CRC screening by colonoscopy could reduce CRC-related incidence and mortality by approximately 18% and 11%, respectively [5]. Although colonoscopy is the most sensitive test for detecting CRC and premalignant lesions, it is both costly and invasive. In addition, its effectiveness in preventing CRC depends on the quality of the colonoscopic procedure [6]. Although training of experienced colonoscopists may contribute to increased polyp detection [7], the learning curve can be steep, and the time cost of training can be high [8,9]. In addition, conventional colonoscopy has a major limitation—up to 27% of adenomatous polyps could be missed due to cognitive and technical capabilities [10].

Artificial intelligence (AI)–assisted colonoscopy, an emerging colonoscopy technology, differs from traditional colonoscopy in that it uses machine learning algorithms to provide physicians with real-time, automated analysis results and highlight potential lesions and polyps [11]. Recently, the development and adoption of AI-assisted colonoscopy have proven successful in identifying and characterizing premalignant or malignant colorectal lesions. A recent systematic review and meta-analysis of randomized controlled trials comparing AI-assisted colonoscopy and conventional colonoscopy demonstrated that an AI-based strategy reduced polyp miss rate by 53% and adenoma miss rate by 50%, respectively. In addition, the AI-assisted technology increased the polyp detection rate, adenoma detection rate (ADR), polyps detected per colonoscopy, and adenomas detected per colonoscopy by 23.8%, 24.2%, 38.8% and 39%, respectively [12]. The authors proposed that future studies should be performed to evaluate the cost-effectiveness of using AI-assisted technology in CRC screening programs, as it has the potential to enhance the impact of CRC screening on reducing mortality.

### Objectives

A comprehensive literature search identified only 2 cost-effectiveness analyses (CEAs) of the use of AI-assisted colonoscopy. They focused on a specific population and compared conventional colonoscopy with AI-assisted colonoscopy as a primary screening test, respectively [13,14]. Previously, we conducted economic evaluations of CRC screening using the fecal immunochemical test (FIT) across various population subgroups and built a statistical model that incorporates different parameters of screening tests to compute different cost-effectiveness outcomes [15]. This study aimed to conduct a comparative CEA of AI-assisted colonoscopy, both as a primary and a secondary screening test within population-based CRC screening programs, and compare it with FIT and conventional colonoscopy.

## Methods

### Ethical Considerations

No ethics approval or exemption or deidentification process was needed for this study.

### Decision Model Framework

**Figure 1:**
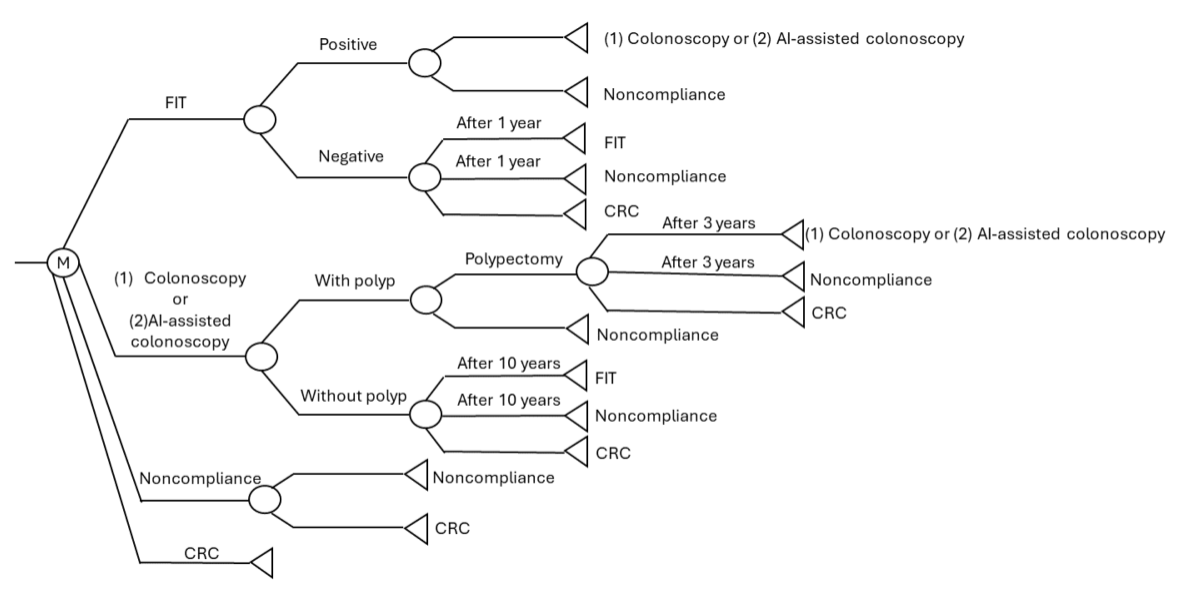
Markov process on strategies using the fecal immunochemical test (FIT) as the primary screening test. AI: artificial intelligence; CRC: colorectal cancer; M: Markov node.

**Figure 2:**
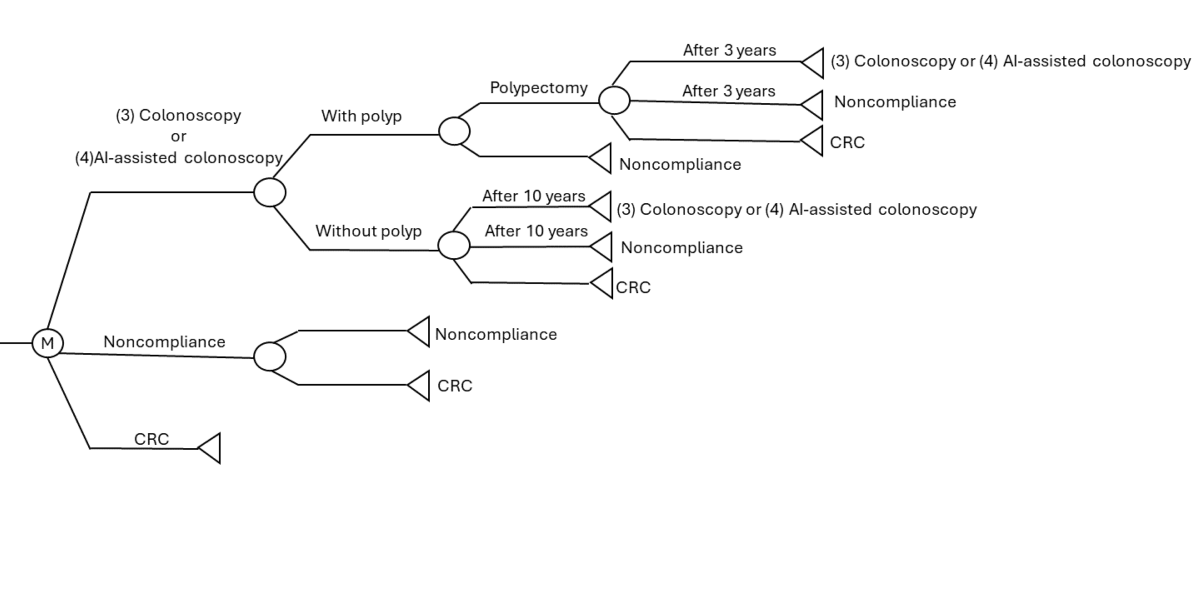
Markov process on strategies using colonoscopy as the primary screening test. The M represents the Markov node. AI: artificial intelligence; CRC: colorectal cancer.

Although some recent studies have recommended CRC screening for individuals aged 45 years to prevent early-onset CRC [16], the age of 50 years is still used as the starting age for CRC screening in many Asian regions, such as Hong Kong, China, and South Korea [17-19]. To better understand the differences between AI-assisted colonoscopy and current CRC screening strategies and their effectiveness, this study modeled a hypothetical population consistent with current clinical practice in Asia, where screening typically begins at the age of 50 years. A hypothetical population of 100,000 average-risk individuals aged 50 years with no history or symptoms of CRC was assumed, and their outcomes were calculated based on assumptions from previous clinical studies conducted in Asian populations (Table 1). Risk factors associated with progression to CRC were included in the assumptions for clinical transition probabilities. The data were entered into a decision analysis algorithm based on a Markov model (Figures 1 and 2). The Markov model is a widely used, powerful tool in health economics and CEA for the evaluation of complex health treatment options over time while incorporating uncertainty. Currently, most guidelines recommend either the FIT followed by conventional colonoscopy or direct colonoscopy as the primary screening strategy [16,17,19]. Given the objective of this study to understand the cost-effectiveness of AI-assisted colonoscopy in CRC screening, we hypothesized that the entire population received four distinct screening strategies, including (1) FIT followed by conventional colonoscopy if the FIT result is positive (FIT+colonoscopy), (2) FIT followed by AI-assisted colonoscopy if the FIT result is positive (FIT+AI-assisted colonoscopy), (3) colonoscopy as the primary screening test, and (4) AI-assisted colonoscopy as the primary screening test. These screening participants were followed up until the age of 75 years. These strategies were compared to a no screening scenario, with reference to a previous CEA [20]. The annual screening intervals for individuals receiving FIT with negative results were set in accordance with current CRC screening guidelines, including those from Hong Kong and the United States [16,17], in the hope that this approach would ensure comparability with existing strategies and provide meaningful information to policymakers.

**Table 1:** Baseline estimates for the screening strategies.

Rate		Estimate (%)	Studies
Sensitivity of FIT^a^ (cutoff value=20 μg/g) in detecting CRC^b^		73	[21]
Specificity of FIT (cutoff value=20 μg/g) in detecting CRC		91.9	[22]
Compliance rate of FIT screening		60	[21]
Compliance rate of colonoscopy screening		98.9	[23]
Compliance rate of colonoscopy screening after a positive result		100	[24]
Polypectomy rate following a positive FIT result		73	[15]
Polypectomy bleeding rate		0.98	[25]
Polypectomy perforation rate		0.08	[25]
Morality due to perforation		0.0029	[25]
Cancer prevented by FIT		21	[26]
Cancer prevented by non-AI^c^ colonoscopy		44.2	[13]
Cancer prevented by AI-assisted colonoscopy		48.9	[13]
**Stage of CRC during diagnosis**			
	1	11.3	[15]
	2	25.4	[15]
	3	32.4	[15]
	4	31	[15]
**Annual mortality of patients with CRC at various stages of disease (1 y)**			
	1	1	[15]
	2	4.5	[15]
	3	8.7	[15]
	5	43	[15]

^a^FIT: fecal immunochemical test.

^b^CRC: colorectal cancer.

^c^AI: artificial intelligence.

### Strategies 1 and 2: FIT+Colonoscopy or AI-Assisted Colonoscopy

Each participant received 1 FIT, with the test repeated yearly among those with negative results. Those with positive FIT results are offered a colonoscopy (strategy 1) or an AI-assisted colonoscopy (strategy 2) for subsequent workup. Participants with normal conventional or AI-assisted colonoscopy will resume FIT examination after 10 years, while those with adenomas removed will receive a surveillance colonoscopy (conventional or AI assisted) arranged every 3 years until there are no more polyps.

### Strategies 3 and 4: Colonoscopy or AI-Assisted Colonoscopy as the Primary Screening Test

Every screening participant received a direct conventional colonoscopy (strategy 3) or an AI-assisted colonoscopy (strategy 4). Those with normal results will receive a repeat conventional or AI-assisted colonoscopy after 10 years, while those with adenomas removed during the index colonoscopy will be offered a conventional or AI-assisted colonoscopy every 3 years until no more polyps are detected. Subsequent surveillance colonoscopy will be scheduled 10 years later after the confirmation of complete removal of adenomas.

### Clinical Transition Probabilities

The transition probabilities constructed into the models were derived from published literature in Asian countries (Table 1). The sensitivity and specificity of FIT in detecting CRC were set at 73% and 91.9%, respectively [21,22]. The compliance rates of FIT and colonoscopy were estimated based on figures from a large Asian population [27]. As the compliance rates of screening tests in Asia vary according to countries, we assumed these compliance rates for the screening tests in the base-case model and tested the possible range of compliance from 10% to 100% in a sensitivity analysis. The compliance with colonoscopy after a positive FIT result was assumed to be 100% [24]. The rate of positive results on FIT was calculated as the sum of true- and false-positive cases. The polypectomy rate following a positive FIT result, bleeding rate, and rate of perforation in colonoscopy were assumed to be 73%, 0.98% and 0.08%, respectively [25]. The mortality rate associated with perforation was determined to be 0.0029%, as reported in a systematic review [25]. The selection of screening intervals was guided by assumptions derived from a previous CEA [20].

In the absence of screening, the population was expected to develop CRC based on age-specific annual incidence rates derived from Hong Kong, China [26]. More parameters used in the model were as follows: the age-specific incidence of CRC for the general population without screening was retrieved from the Hong Kong Cancer Registry, which were 55.9, 89.8, 137, 200, 253.2, and 330.5 per 100,000 for quinquennial age range from 50 to 75 years (Hong Kong Cancer Registry Hong Kong cancer statistics available online [28]. The mortality rate for each age was based on a reference.

The detection of CRC at earlier stages improves overall survival. According to a network meta-analysis, the use of FIT can reduce CRC incidence by 21% [29]. The reduction in CRC cases after annual screening by colonoscopy was assumed to be 51.5% from a territory-wide CRC screening program in Hong Kong and a previous CEA performed by our team [23].

The annual mortality rates of CRC diagnosed at different stages were determined based on data from the Hong Kong Cancer Registry [26]. Patients diagnosed with stage 1 and stage 2 CRC were treated with surgery alone with the intent to cure (recurrence free for at least 3 to 5 years). Patients diagnosed with stage 3 CRC were offered adjuvant chemotherapy after surgery. Approximately 70% of the patients with CRC are expected to be cured, while the remaining tend to develop recurrent disease. Patients diagnosed with stage 4 CRC were either offered palliative colorectal surgery followed by palliative chemotherapy, direct chemotherapy alone, or no treatment. Among patients with stage 4 CRC, approximately 50% required further surgery to address liver metastasis [20]. Five common regimens are available, including 5-fluorouracil combined with folinic acid , folinic acid, fluorouracil and irinotecan, folinic acid, fluorouracil and oxaliplatin (FOLFOX), Avastin, and Cetuximab. Among these, 5-fluorouracil combined with folinic acid is considered the first-line agent in both adjuvant and metastatic settings. For the base-case model in this study, it was assumed that patients with stage 3 CRC receive FOLFOX for 6 months, while those with stage 4 CRC receive FOLFOX combined with bevacizumab (Avastin; Roche Genentech Inc) for 10 months. These regimens were chosen as they represented the current standard and most effective treatments in adjuvant and metastatic settings [30]. Once patients were cured of CRC, the model did not account for follow-up surveillance or management of possible cancer recurrence.

### Cost Estimates

The decision model included direct costs of screening tests; costs of investigation and CRC staging, including computed tomography and positron emission tomography scans; costs of cancer treatments, such as surgery and chemotherapy; and hospitalization costs (Table 2). In this study, the investment cost of AI-assisted colonoscopy was excluded from the cost estimate because its 1-time cost could be as low as US $19 and, combined with the nature of reusability, does not impact the robustness of the study results [14]. In addition, similar to previous CEA studies [13], the cost of AI-assisted colonoscopy in this study was assumed to be consistent with that of conventional colonoscopy, as there were no real-world data on the cost of AI-assisted colonoscopy, particularly in the Asia region.

**Table 2:** Estimates for the costs based on different screening strategies and treatment methods.

Cost item		Baseline value (US $)	Studies
One kit of FIT^a^		19	[21]
Colonoscopy		1259	[20,30]
Consultation fees		96	[20,30]
Bleeding		3320	[20,30]
Histopathological examination		142	[20,30]
Perforation		10,790	[20,30]
**Treatment for stage 1 CRC^b^**		17,071	[20,30]
	Diagnosis	6091	[20,30]
	Treatment	10,377	[20,30]
	Follow-up (in 9 days)	603	[20,30]
**Treatment for stage 2 CRC**		19,755	[20,30]
	Diagnosis	6091	[20,30]
	Treatment	13,061	[20,30]
	Follow-up (in 9 days)	603	[20,30]
**Treatment for stage 3 CRC**		26,883	[20,30]
	Diagnosis	6091	[20,30]
	Treatment (FOLFOX^c^ for 6 months)	20,189	[20,30]
	Follow-up (in 9 days)	603	[20,30]
**Treatment for stage 4 CRC**		45,115	[20,30]
	Diagnosis	6091	[20,30]
	Treatment (FOLFOX+bevacizumab for 10 months)	38,422	[20,30]
	Follow-up (in 9 days)	603	[20,30]

^a^FIT: fecal immunochemical test.

^b^CRC: colorectal cancer.

^c^FOLFOX: folinic acid, fluorouracil and oxaliplatin.

The costs per kit for FIT, AI-assisted colonoscopy, and conventional colonoscopy were included as direct costs. The costs of diagnosis and preoperative assessment included health services such as colonoscopy with or without biopsy, histopathological examination, carcinoembryonic antigen-level testing, computed tomography contrast scan for abdomen and pelvis, ultrasound of abdomen, magnetic resonance imaging contrast scan for pelvis, positron emission tomography scan, and outpatient specialist clinic. The treatment costs included the costs of surgery and chemotherapy, and the cost of 6 months of FOLFOX and 10 months of FOLFOX+bevacizumab was included for patients with stage 3 and stage 4 CRC, respectively. Follow-up costs included outpatient consultations, hospitalization due to colonoscopy or polypectomy complications (bleeding or perforation), routine inpatient care, and the cost of disposable devices. In addition, we assumed an average length of stay of 9 days for patients undergoing CRC surgery [30]. The costs of repeat visits and blood transfusions were excluded from the analysis. Indirect costs, such as transportation to the hospital or productivity loss because of absence from work, were also excluded for simplicity. All future costs related to CRC screening and care as well as all future life years saved through screening were discounted at an annual rate of 3% [31].

### CEA Evaluation

The effectiveness of screening was measured in terms of life years saved through the prevention of CRC and improved survival due to earlier diagnosis. Years of life lost were estimated using the Hong Kong standard life table. Age-dependent proportions of patients dying prematurely due to CRC were used to calculate life years lost, which were accumulated for each cycle over the entire expected lifetime. The number of life years saved because of screening corresponds to the difference in life years lost to cancer-related deaths between a Markov model with screening and one without screening. The key outcome of this study was the incremental cost-effectiveness ratio (ICER) between the screening strategies, that is, the cost difference divided by the difference in effectiveness between the strategies. ICER quantifies the additional cost required for a life year saved.

### Sensitivity Analysis

As health care costs vary across different Asian countries, sensitivity analyses of the ICER were conducted to assess their robustness across different intervals of key parameters, including compliance rates of screening tests, the sensitivity of FIT, and the cost of colonoscopy. One-way sensitivity analyses of ICER between different screening strategies over the possible range of model variables were conducted. Compliance rates for initial, repeated, and follow-up screenings were assumed to be the same. In cases where the results were not robust, threshold values at which the conclusions would change were identified and reported. All calculations were simulated by using Microsoft Excel spreadsheets.

## Results

### Cost-Effectiveness Comparison of Various Strategies

The outcomes of the 4 screening strategies and a no screening scenario are shown in Table 3, where life years saved and the associated costs reflect the effect of an annual discount rate of 3%. Without screening, a cohort of average-risk participants aged 50 years had 3233 cases of CRC and a loss of 5635 cancer-related life years. Screening using FIT+colonoscopy, FIT+AI-assisted colonoscopy, direct conventional colonoscopy, and direct AI-assisted colonoscopy prevented 3.7%, 4.1%, 41.9%, and 46.4% of CRC cases, respectively. Screening with conventional colonoscopy and AI-assisted colonoscopy resulted in more life years saved and greater mortality reduction than FIT-based strategies but at a higher cost. The treatment cost for advanced-stage CRC (stages 3 and 4) was substantially higher. Hence, early detection through screening reduced both cancer mortality and treatment expenditures.

**Figure 3:**
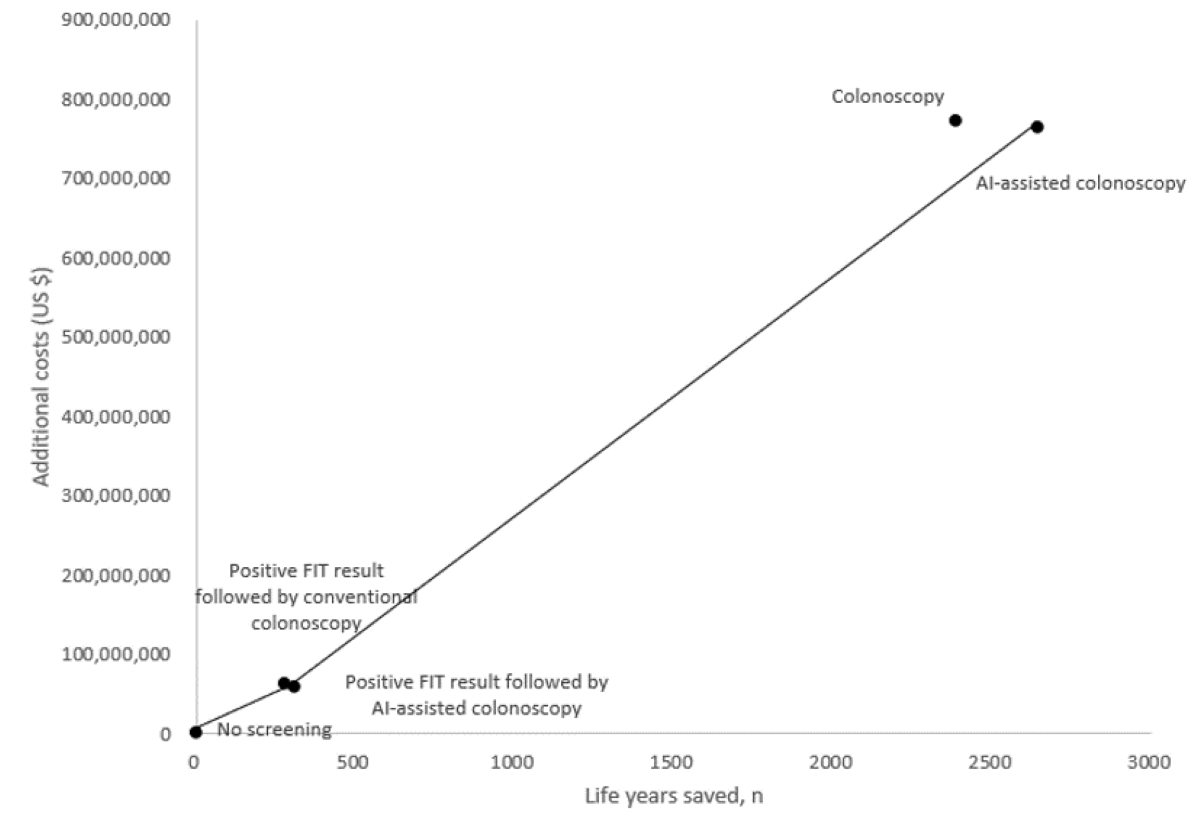
Cost-effectiveness analysis for the screening tests. AI: artificial intelligence; FIT: fecal immunochemical test.

The total costs for managing CRC among screening participants who received FIT+colonoscopy (US $264 million), FIT+AI-assisted colonoscopy (US $263 million), colonoscopy alone (US $712 million), and AI-assisted colonoscopy (US $702 million) are reported in Table 3. Compared with the no screening strategy, the ICERs of FIT+colonoscopy, FIT+AI-assisted colonoscopy, colonoscopy alone, and AI-assisted colonoscopy were US $138,539, US $122,539, US $203,929, and US $180,444, respectively (Figure 3; Table 3). Therefore, FIT+AI-assisted colonoscopy emerged as the most cost-effective strategy for CRC screening and prevention.

**Table 3:** Outcomes of a cohort of average-risk individuals aged between 50 and 75 years with various screening strategies for colorectal cancer (CRC; N=100,000).

Screening method				No screening		FIT^a^ positive result followed by a conventional colonoscopy		FIT positive result followed by AI^b^ colonoscopy		Direct colonoscopy		Direct AI-assisted colonoscopy
CRC cases, n				3233		3113		3100		1879		1733
Total loss of cancer-related life years (y)				5635		5355		5327		3251		2996
Cases of CRC prevented, n				0		120		132		1354		1499
Proportion of CRC cases prevented (%)				0		3.7		4.1		41.9		46.4
Life years saved (y)				0		280		308		2384		2639
**Number of procedures**												
	FIT		0		236,883		236,897		0		0	
	Colonoscopy		0		36,635		36,652		508,957		509,216	
	Diagnostic (without polypectomy)		0		9891		9896		141,505		141,505	
	Therapeutic (with polypectomy)		0		26,744		26,756		367,452		367,638	
**Number of complications**												
	Bleeding		0		73		73		1018		1018	
	Perforations		0		29		29		403		403	
**Costs (US $)**												
	FIT		0		4,246,988		4,247,152		0		0	
	Colonoscopy		0		40,523,900		40,539,212		525,693,338		525,902,136	
	Polypectomy		0		3,097,673		3,098,843		39,774,128		39,789,925	
	Bleeding		0		973,052		973,420		12,622,848		12,627,861	
	Perforations		0		258,157		258,254		3,312,081		3,313,396	
	**Care of CRC**											
		Stage 1, US $	4,050,827		3,876,144		3,858,423		2,346,109		2,163,463	
		Stage 2, US $	14,828,981		14,183,061		14,117,550		8,585,039		7,916,156	
		Stage 3, US $	36,148,483		34,556,089		34,394,631		20,918,513		19,287,277	
		Stage 4, US $	170,486,718		162,588,121		161,788,438		98,476,572		90,769,075	
		Total, US $	225,515,010		264,303,185		263,275,923		711,728,628		701,769,288	
Total costs per life year saved (US $)				—^c^		944,008		854,367		298,515		265,888
Additional costs (US $)^d^				0		38,788,175		37,760,914		486,213,619		476,254,278
ICER^e^ vs no screening				—		138,539		122,539		203,929		180,444
ICER vs FIT positive result followed by colonoscopy				—		—		–36,462 (dominated)		212,629		185,417
ICER vs FIT positive result followed by colonoscopy AI				—		—		—		216,009		188,099
ICER vs colonoscopy				—		—		—		—		–39,040 (dominated)

^a^FIT: fecal immunochemical test.

^b^AI: artificial intelligence.

^c^Not applicable

^d^The additional cost was calculated by comparing the total cost of the strategy to the no screening strategy.

^e^ICER: incremental cost-effectiveness ratio.

### Sensitivity Analysis of ICER Across Strategies

**Figure 4:**
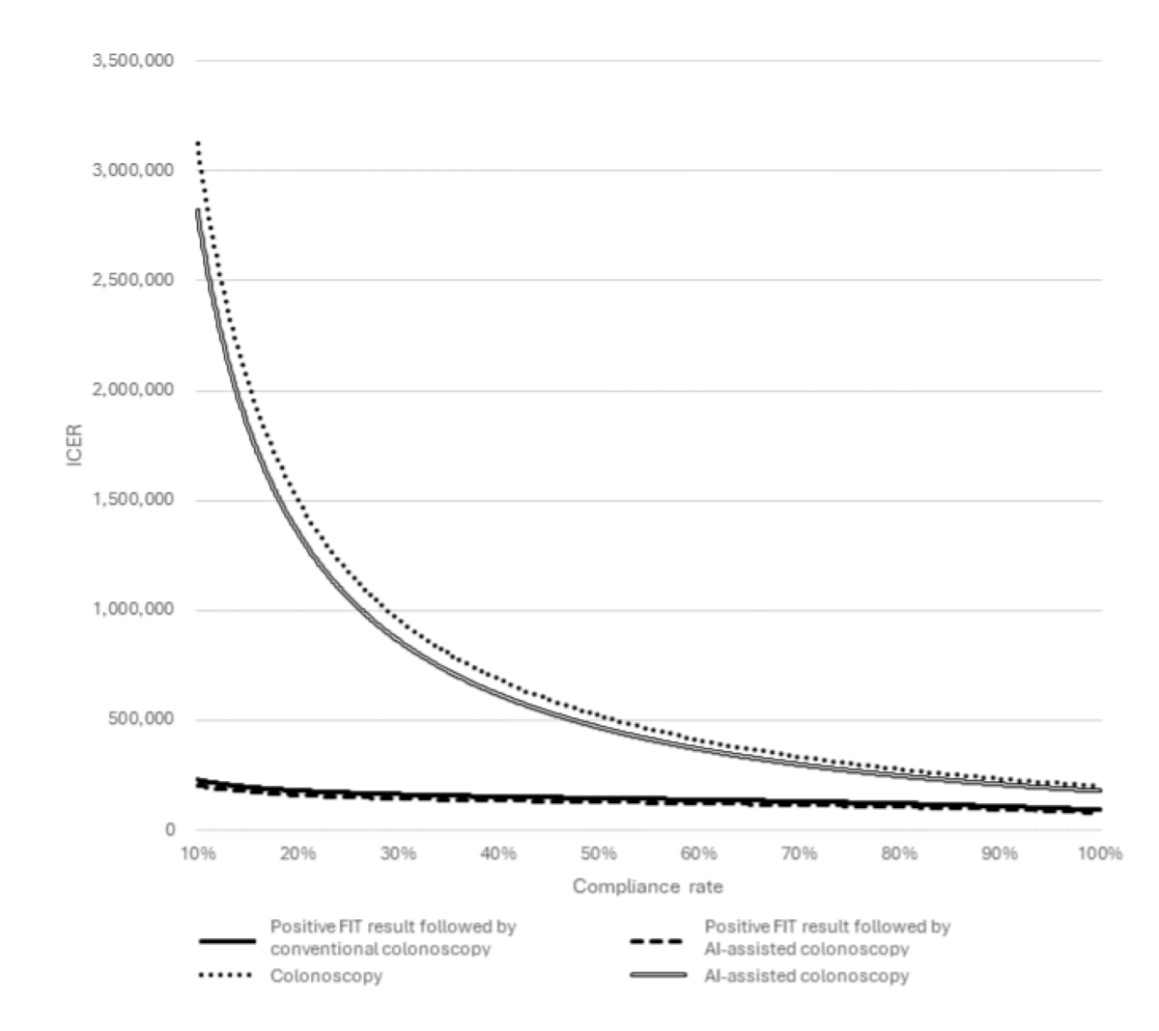
One-way sensitivity analysis on the compliance rate of screening tests. AI: artificial intelligence; FIT: fecal immunochemical test; ICER: incremental cost-effectiveness ratio.

ICERs were influenced by the compliance rate. One-way sensitivity analysis was performed, testing a range of compliance rates (Figure 4). Reduced compliance rates increased ICERs, indicating higher costs per life year saved. Yet, ICER remained the lowest for the FIT+AI-assisted colonoscopy strategy as compared to other strategies.

**Figure 5:**
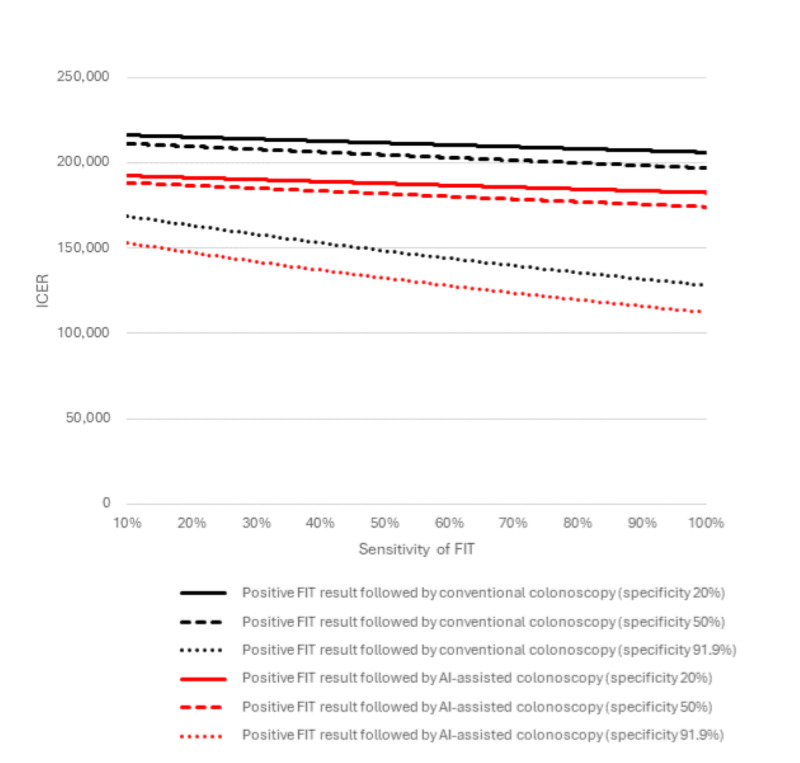
Two-way sensitivity analysis on the sensitivity and specificity of FIT. AI: artificial intelligence; FIT: fecal immunochemical test; ICER: incremental cost-effectiveness ratio.

The performance of AI-assisted colonoscopy determined its cost-effectiveness compared to other screening strategies. The ICER for AI-assisted colonoscopy increased as the specificity of the FIT decreased from 91.9% to 20% (Figure 5). The results indicated that higher sensitivity and specificity improved the cost-effectiveness of AI-assisted colonoscopy. In contrast, a decrease in the sensitivity of FIT led to a small increase in ICER. Regardless of changes in FIT sensitivity, the ICER of FIT followed by AI-assisted colonoscopy remained lower than that of FIT followed by conventional colonoscopy.

**Figure 6:**
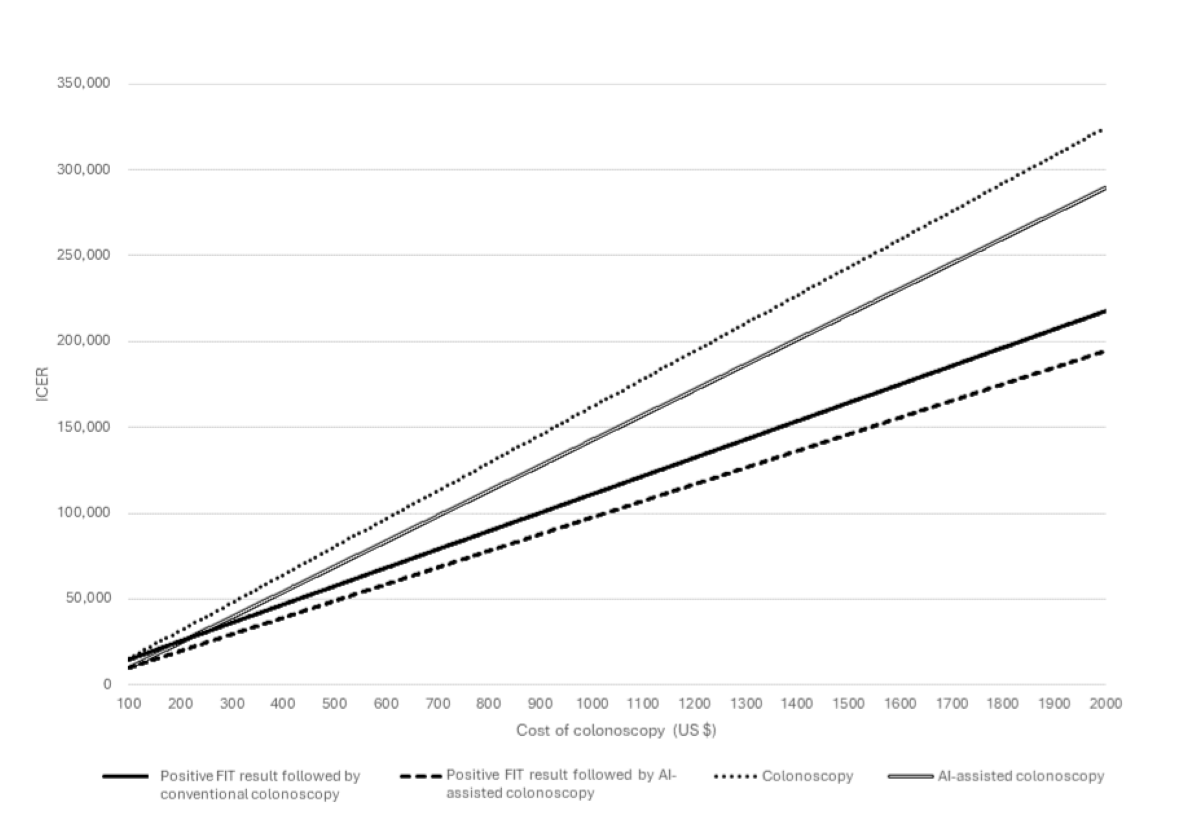
One-way sensitivity analysis on the cost of colonoscopy. AI: artificial intelligence; FIT: fecal immunochemical test; ICER: incremental cost-effectiveness ratio.

Higher treatment costs for CRC resulted in increased ICERs across all strategies. The cost of colonoscopy influenced direct colonoscopy and AI-assisted colonoscopy more than the fecal-based screening strategies. Moreover, the AI-assisted colonoscopy was more cost-effective in patients with a positive FIT result and direct colonoscopy method (Figure 6). The difference between ICERs of different strategies was noticeable; therefore, FIT+AI-assisted colonoscopy remained the most cost-effective approach compared to no screening, FIT positive result followed by conventional colonoscopy, and direct conventional colonoscopy.

## Discussion

### Principal Findings

The findings of this study highlighted the cost-effectiveness of different screening strategies and provided an intuitive way to compare different strategies through quantitative outcomes such as ICER. These findings can provide further evidence to inform policymakers and improve the existing CRC screening strategies, especially in Asian countries.

This study aimed to evaluate the cost-effectiveness of various CRC screening methods, including no screening, FIT+colonoscopy, FIT+AI-assisted colonoscopy, direct colonoscopy, and direct AI-assisted colonoscopy. The findings indicated that FIT screening followed by an AI-assisted colonoscopy approach was the most cost-effective strategy with the lowest ICER compared to other methods. Furthermore, AI-assisted colonoscopy demonstrated lower total costs per life year saved compared to conventional colonoscopy. Overall, these results highlight the cost-effectiveness of the FIT+AI-assisted colonoscopy strategy as a primary screening approach for CRC screening.

In 2024, Hong Kong had approximately 2.8 million individuals aged between 50 and 74 years [32]. Our findings indicate that the FIT+AI-assisted colonoscopy strategy could potentially prevent an additional 4120 CRC cases and save 7245 more life years compared to conventional colonoscopy. Furthermore, the total cost per life year saved could be reduced by approximately US $927,000 when compared to conventional colonoscopy. The FIT+AI-assisted colonoscopy strategy for CRC screening appears to be the most cost-effective strategy with a better balance in cost and life years saved in Hong Kong.

### Potential Benefits and Economic Implications of Incorporating AI-Assisted Colonoscopy Into CRC Screening Programs

Integrating AI systems into colonoscopy does not necessarily require significant additional costs. The findings of this study reveal that AI-assisted colonoscopy has the lowest additional costs among all listed screening methods, which aligns with other CEA studies [14]. Moreover, once AI algorithms are developed and validated, they can be incorporated into existing endoscopy systems without requiring major changes to hardware or infrastructure. In the long run, the actual cost is expected to decrease due to a reduction in CRC incidence, driven by an increase in ADR, and potentially, fewer expert endoscopists will be required. This makes AI-assisted colonoscopy a relatively cost-effective screening tool compared to other advanced imaging techniques. While the advantages of AI-assisted colonoscopy are evident, further large-scale clinical studies are essential to validate its effectiveness [33]. In addition, our findings point to the fact that FIT followed by AI-assisted colonoscopy for screening can save more lives at the lowest additional cost, demonstrating its potential as an alternative to existing CRC screening measures. However, its implementation in the real world may require additional evidence-based support. Some studies suggest that AI-assisted colonoscopy software currently on the market may cost as little as US $19 [14] and can be used with hardware support in standard workstation-level configurations [11]. However, there is insufficient research to support the feasibility of AI-assisted colonoscopy in resource-constrained areas. Therefore, we recommend that future studies investigate the application of AI-assisted colonoscopy in resource-constrained countries.

### Comparison With Previous CEA Studies on AI-Assisted Colonoscopy

Previous CEA studies have shown positive outcomes regarding the implementation of AI in colonoscopy, which align with the findings of this study. Jootun et al [34] conducted a CEA in Spain involving a cohort of 1000 patients and demonstrated an increase in average life years, quality-adjusted life years (QALYs), and cost-effectiveness of AI-assisted colonoscopy compared to conventional colonoscopy. Similarly, Barkun et al [13] conducted a study in Canada, focusing on the cost-effectiveness of AI-assisted colonoscopy. The study also observed significant improvements in terms of life years saved, QALYs gained, and the ICER. Areia et al [14] conducted a study involving 100,000 patients in the United States and reported similar results. Furthermore, multiple meta-analyses and cost-effectiveness studies have consistently shown positive outcomes for AI-assisted colonoscopy [35]. These studies, conducted globally in countries with different levels of development, support the generalizability of the findings and highlight the beneficial outcomes of AI assistance. While several studies have focused on the benefits of and improvements offered by AI-assisted colonoscopy in Hong Kong, there has been a lack of research on the costs of implementing AI in CRC screening. This study adds value by investigating the cost-effectiveness of AI-assisted colonoscopy compared to existing screening strategies, which provides insights that can inform future CRC screening policies in the Asia-Pacific region and beyond.

### Strengths of AI-Assisted Colonoscopy When Compared With Conventional Colonoscopy

This study evaluated the cost-effectiveness of AI-assisted colonoscopy over conventional colonoscopy, and there were additional benefits of using AI-assisted colonoscopy over conventional colonoscopy apart from cost considerations. ADR is inversely associated with interval postcolonoscopy CRC in different settings [6]. A previous meta-analysis reported that AI-assisted colonoscopy improved ADR by approximately 10% [36]. Another study compared conventional colonoscopy and AI-assisted colonoscopy across 6 referral centers in China and found that AI-assisted colonoscopy significantly increased overall ADR, advanced ADR, and ADR among both expert and nonexpert endoscopists [37]. AI algorithms could help analyze real-time video feeds during colonoscopy procedures, assisting endoscopists by flagging suspicious areas that might otherwise be missed by the naked eye, thereby reducing human error. This can help improve the overall sensitivity of the procedure and increase the detection rate of polyps and lesions.

A prospective study revealed that AI-assisted colonoscopy had a significantly longer withdrawal time compared to conventional colonoscopy, approximately 7 minutes versus 5.4 minutes, respectively [38]. The US Multi-Society Task Force on CRC recommends a withdrawal time of at least 6 minutes to increase ADR [39]. In conventional colonoscopy, shorter withdrawal times may be attributed to busy clinic schedules and the lack of workforce. Although this accelerates the withdrawal time, it can compromise withdrawal stability. AI-assisted colonoscopy has a longer withdrawal time. However, the AI system controls the inspection speed and time, thereby improving ADR and reducing the likelihood of false-negative results.

### Limitations of This Study

This cost-effectiveness study relied on a hypothetical population and made certain assumptions regarding the evaluation of various CRC screening methods. However, there are several limitations of this study that need to be recognized. First, given that the assumptions of this study are based on data relevant to Asian populations, some factors such as the availability of health care resources and health behavior in different regions may affect the applicability of the findings. In addition, we recognize the importance of incorporating actual adherence rates based on studies involving a larger population size in future research. It is crucial to conduct further studies to assess the actual costs involved in the development and implementation of AI systems and the estimated cost-effectiveness of AI-assisted colonoscopy. Second, our study did not compare the cost-effectiveness of different surveillance strategies in this study, which may also be important for the implementation of CRC screening at the population level. Health care professionals should present various CRC screening options to patients, taking into account their specific needs and circumstances. These considerations highlight the need for ongoing research and personalized approaches to CRC screening. Third, this study did not measure the QALYs and the cost-effectiveness acceptability of various screening strategies, primarily due to the lack of robust data on quality-of-life indicators and the willingness-to-pay threshold specific to the Hong Kong or Asian population. However, we believe these variables have significant potential for future research, yielding valuable insights into the feasibility of different CRC screening strategies. Therefore, we recommend that future studies address these gaps by integrating real-world adherence data, evaluating surveillance strategies, and incorporating QALYs and acceptability analyses to better inform evidence-based CRC screening policy.

### Conclusions

This study demonstrated that FIT followed by AI-assisted colonoscopy was the most cost-effective strategy for an Asian population, as it dominated ICER analysis and incurred the lowest additional costs among the 4 strategies. In contrast, using direct conventional or AI-assisted colonoscopy as a primary screening method resulted in more life years saved and a greater reduction in mortality compared to FIT-based strategies at the expense of higher total costs per life year saved and higher additional costs.

## Abbreviations

ADR: adenoma detection rate
AI: artificial intelligence
CEA: cost-effectiveness analysis
CRC: colorectal cancer
FIT: fecal immunochemical test
FOLFOX: folinic acid, fluorouracil and oxaliplatin
ICER: incremental cost-effectiveness ratio
QALY: quality-adjusted life year

## Data Availability

The datasets generated or analyzed during this study are available from the corresponding author on reasonable request.
